# Lactobacillus Discitis/Osteomyelitis in an Intravenous Drug Abuser

**DOI:** 10.7759/cureus.9219

**Published:** 2020-07-16

**Authors:** Yasmeen Obeidat, Mohamed S Suliman, Michael Mullins, Elizabeth Saunders

**Affiliations:** 1 Internal Medicine, Marshall University Joan C. Edwards School of Medicine, Huntington, USA

**Keywords:** osteomyelitis, lactobacillus, intravenous drug user, disciitis, clindamycin

## Abstract

Vertebral osteomyelitis is usually secondary to hematogenous seeding from direct inoculation during spinal surgery or from adjacent soft tissue infection; the most common organism being Staphylococcus aureus. We present a case of a 31-year-old male who was found to have vertebral osteomyelitis secondary to Lactobacillus species. The patient with a past medical history significant for hepatitis C, intravenous (IV) drug use, and nicotine dependence presented with severe back pain that started one month ago. His pain was located in the middle and lower back, radiating to his abdomen, and both lower extremities. The patient admitted to abusing IV heroin daily and sharing needles with his fiancée. CT of the abdomen and pelvis with contrast revealed marked irregularity of the endplates at the L3-L4 level and mild irregularity of the endplates at the L4-L5 level suggestive of osteomyelitis/discitis with no evidence of a paraspinal fluid collection. Core biopsy of the superior endplate of L4 and adjacent disc material was done and sent for microbiology and pathology review. His bone culture came back positive for Lactobacillus species; however, blood cultures remained negative. Clinical improvement was noted after starting antibiotics, and the patient was discharged on six weeks of oral clindamycin. When thinking of Lactobacillus, a simple probiotic comes to mind, clinicians need to be more vigilant in recognizing its different strains as possible infectious microorganisms. As described in our case, and other cases of bacteremia secondary to Lactobacillus, Lactobacillus should no longer be regarded as merely a contaminant.

## Introduction

Vertebral osteomyelitis is usually secondary to hematogenous seeding from direct inoculation during spinal surgery or from adjacent soft tissue infection; the most common organism being Staphylococcus aureus [1]. Its incidence has been reported at 2.4 cases per 100,000 population [2]. With the narcotic abusing population not practicing sterile technique, vertebral osteomyelitis may occur as an infectious complication of intravenous (IV) drug use, almost exclusively in heroin users [3]. It has been reported that the most common pathogenic organisms involved in vertebral osteomyelitis in IV drug users are gram-negative rods [3]. We present a case of a 31-year-old male who was found to have vertebral osteomyelitis secondary to Lactobacillus species.

## Case presentation

**Figure 1:**
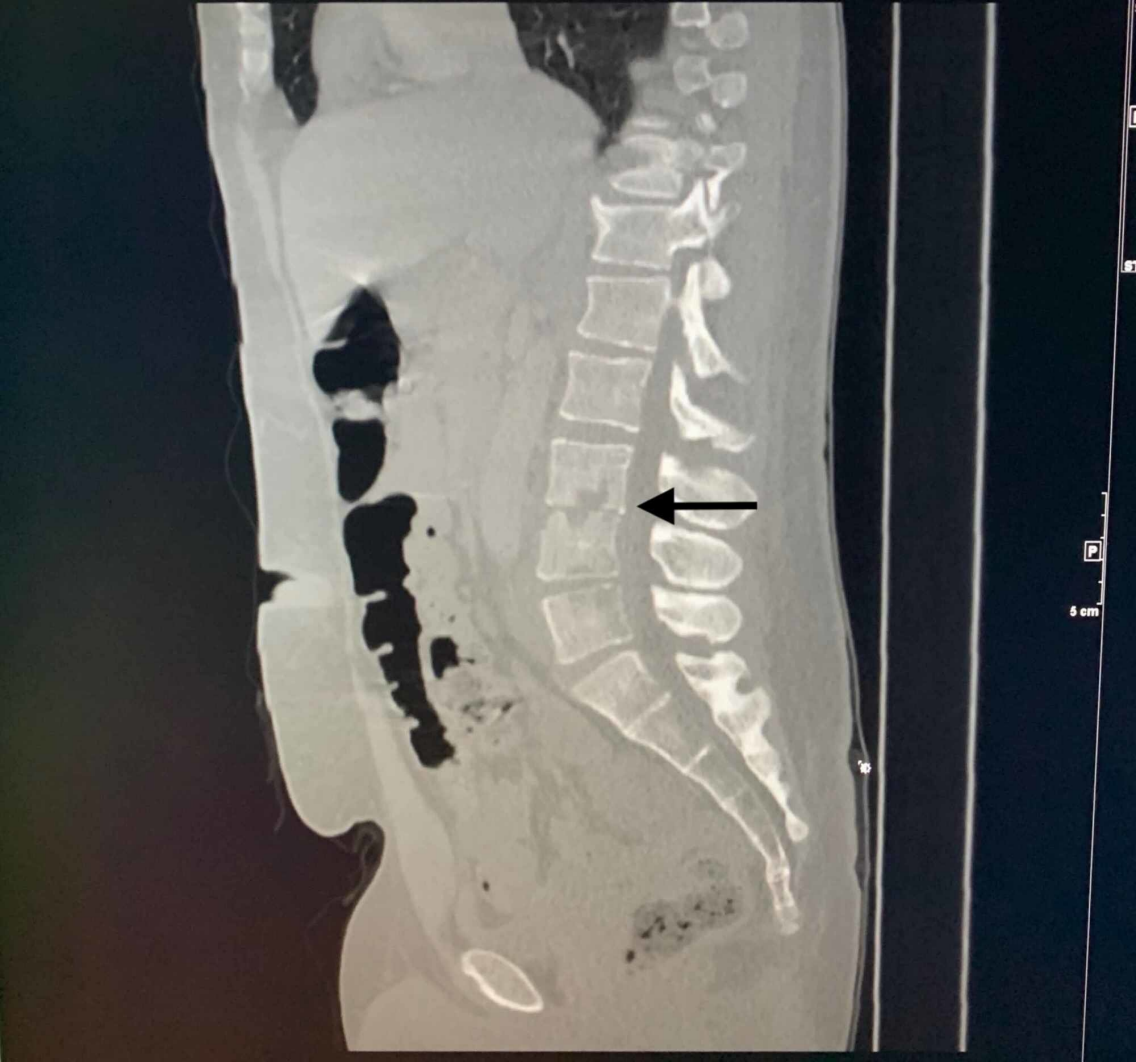
CT of the abdomen and pelvis with contrast showing marked irregularity of the endplates at the L3-L4 level and mild irregularity of the endplates at the L4-L5 level suggestive of osteomyelitis/discitis.

A 31-year-old male with a past medical history significant for hepatitis C, IV drug use, and nicotine dependence presented with severe back pain that started one month ago. He reported that the pain started suddenly while he was trying to get out of his vehicle and progressed to the point where he could not to lay flat or sit down. His pain was located in the middle and lower back, radiating to his abdomen, and both lower extremities. He denied any numbness, tingling, weakness, or decreased sensation of the lower extremities. He also denied urinary/stool incontinence, fever, or chills. There was no pertinent prior history of back trauma or surgery. The patient admitted to abusing IV heroin daily and sharing needles with his fiancée. Upon admission to our facility, vital signs were within normal range except for a heart rate of 115 beats per minute (bpm). On physical examination, significant tenderness was noted at the middle and lower spine. No obvious bony deformity or skin abnormalities were noted. Neurological examination was normal except for decreased power 3/5 in bilateral lower extremities. Extensive laboratory work-up was done revealing a white blood cell count of 10.7 x 10^9^ cells per liter, hemoglobin 12.8 g/dl, with the rest of the work-up within normal range including lactic acid, urinalysis, creatinine, glucose, liver function, and serum electrolytes. CT of the abdomen and pelvis with contrast revealed marked irregularity of the endplates at the L3-L4 level and mild irregularity of the endplates at the L4-L5 level suggestive of osteomyelitis/discitis with no evidence of a paraspinal fluid collection (Figure 1).

Core biopsy of the superior endplate of L4 and adjacent disc material was done and sent for microbiology and pathology review. In addition, blood cultures were obtained. The patient was started on vancomycin and piperacillin-tazobactam. His bone culture came back positive for Lactobacillus species; however, blood cultures remained negative. Infectious disease was consulted and recommended switching antimicrobial coverage to ampicillin. He then developed a rash prompting a switch from ampicillin to clindamycin. Clinical improvement was noted after starting antibiotics, and the patient was discharged on six weeks of oral clindamycin.

## Discussion

Vertebral osteomyelitis is most often from hematogenous seeding in those undergoing spinal surgery or patients with contagious spread from adjacent soft tissue infection; the most common microorganism implicated being Staphylococcus aureus followed by Escherichia coli [1]. Different populations, however, are susceptible to different strains and mechanisms of infection and in the IV drug using population specifically, Vertebral osteomyelitis is secondary to inoculation without the sterile technique, with the most common organisms reported being gram-negative rods [3]. ﻿In the analysis of a 14 case series, back pain was reported as the most common complaint [4]. Lactobacilli are gram-positive, non-spore-forming rods or coccobacilli that are strictly fermentative, aerotolerant or anaerobic, aciduric or acidophilic, and have complex nutritional requirements [5]. In humans, they colonize the oral cavity, gastrointestinal (GI) tract, and vagina, and generally have little or no virulence with bacteremia due to lactobacilli among immunocompetent patients being rare [6]. Despite its rarity and low virulence, Lactobacillus has been reported as the causative pathogen in endocarditis and bacteremia with the species casei and rhamnosus being the most common; these isolates tended to be most sensitive to erythromycin and clindamycin [7]. The pathogenic role of Lactobacillus was further explored when the Lactobacillus casei/paracasei strain was isolated as an exclusive agent of spondylodiscitis, which to the authors' knowledge was one of the first few cases reported [8]. In our patient, once Lactobacillus was identified from bone culture, oral antibiotic treatment with clindamycin was pursued and served as sufficient therapy which is consistent with the literature.

## Conclusions

When thinking of Lactobacillus, a simple probiotic comes to mind, clinicians need to be more vigilant in recognizing its different strains as possible infectious microorganisms. As described in our case, and other cases of bacteremia secondary to Lactobacillus, Lactobacillus should no longer be regarded as merely a contaminant. Further research may be warranted. 
